# Effect of intensive versus standard blood pressure control on cardiovascular outcomes: a meta-analysis of randomized controlled trials

**DOI:** 10.1080/07853890.2026.2662627

**Published:** 2026-04-30

**Authors:** Qiu Gao, Yanni Ji

**Affiliations:** Department of Cardiology, Yixing People’s Hospital, Yixing, Jiangsu Province, China

**Keywords:** Hypertension, blood pressure, cardiovascular outcomes, meta-analysis

## Abstract

**Background:**

This meta-analysis systematically evaluated the impact of intensive (typically targeting systolic BP [SBP] <120 or <130 mmHg) versus standard (typically targeting SBP <140 mmHg) blood pressure control on cardiovascular outcomes in patients with hypertension.

**Materials and methods:**

Relevant randomized controlled trials (RCTs) published up to May 2025 were identified through systematic searches of PubMed, Embase, Web of Science, and the Cochrane Library. A random-effects model was used to calculate pooled relative risks (RRs) with 95% confidence intervals (CIs).

**Results:**

Thirty-one RCTs involving a total of 156,933 participants (mean age range of trial populations: 36.6 to 83.6 years; proportion of male participants: 34.5% to 69.4%; follow-up duration: 1.8 to 19.3 years) were included in the final meta-analysis. Compared to standard blood pressure control, intensive control significantly reduced the risk of major cardiovascular events (RR: 0.80; 95% CI: 0.75–0.84; *p* < 0.001), myocardial infarction (RR: 0.83; 95% CI: 0.76–0.91; *p* < 0.001), stroke (RR: 0.76; 95% CI: 0.70–0.82; *p* < 0.001), all-cause mortality (RR: 0.87; 95% CI: 0.83–0.92; *p* < 0.001), and cardiac death (RR: 0.79; 95% CI: 0.73–0.86; *p* < 0.001). Sensitivity analyses confirmed the robustness of these findings. Additionally, the treatment effects varied by sample size, male proportion, smoking prevalence, diabetes status, and follow-up duration.

**Conclusion:**

Intensive blood pressure control (typically targeting SBP <120 or <130 mmHg) is strongly associated with a reduced risk of cardiovascular events. These findings support adopting lower blood pressure targets in clinical practice while emphasizing the need for individualized patient assessment.

## GRAPHICAL ABSTRACT

**Figure:**
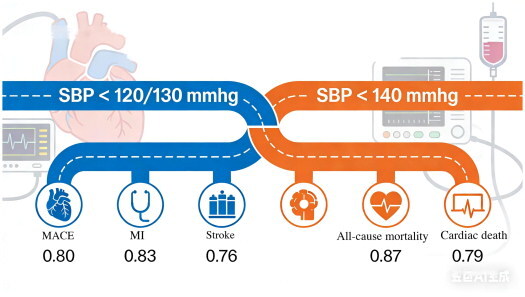

## Introduction

Hypertension is a major global public health challenge and the leading modifiable risk factor for the development and progression of cardiovascular disease (CVD) [1]. Among Chinese adults, the prevalence of hypertension rose from 9.6% in 1982 to 28.6% in 2015, increasing in both men (from 10.9% to 31.3%) and women (from 8.2% to 25.9%) over the same period [2]. Beyond elevated blood pressure readings, hypertension causes sustained damage to critical target organs, including the heart, brain, kidneys, and blood vessels, substantially increasing the risk of major complications such as myocardial infarction, stroke, heart failure, and end-stage renal disease [3–5]. As a result, it threatens individual health and longevity, and imposes a significant burden on healthcare systems worldwide.

Although the dangers of hypertension are well established, and blood pressure-lowering therapy is widely recognized as a cornerstone of cardiovascular prevention, debate persists regarding the optimal blood pressure target. Traditional ‘standard’ control strategies typically recommend conservative systolic blood pressure goals, reflecting a cautious approach that weighs cardiovascular benefits against the risks of overtreatment, such as hypotension, electrolyte imbalances, and transient declines in renal function [6]. In contrast, recent studies have supported an ‘intensive’ control paradigm that advocates for more aggressive management to further reduce residual cardiovascular risk [7,8]. This divergence in treatment philosophy is evident in the conflicting recommendations of current clinical guidelines. Such inconsistencies underscore the gaps and uncertainties in the evidence base and create challenges for clinicians when determining treatment goals and therapeutic intensity in everyday practice [9].

Randomized controlled trials (RCTs), considered the gold standard for evaluating the efficacy and safety of interventions, are essential for resolving this debate. However, individual RCTs often face limitations, including small sample sizes, narrow inclusion criteria, short follow-up periods, heterogeneity in study design, and limited statistical power. These constraints may reduce the generalizability of their findings and lead to inconsistent results. Therefore, synthesizing existing high-quality evidence through a meta-analysis is crucial to overcoming these limitations and generating more comprehensive and reliable conclusions.

In this study, we conducted a meta-analysis comparing the effects of intensive versus standard blood pressure control—as defined by the respective trial protocols, where intensive strategies typically aimed for lower systolic targets (e.g. <120 or <130 mmHg) compared to standard strategies (typically <140 mmHg)—on cardiovascular outcomes in patients with hypertension. Our aim is to provide high-quality, evidence-based insights to help resolve ongoing controversies surrounding blood pressure targets and offer a robust scientific foundation for optimizing clinical decision-making in hypertension management.

## Methods

### Search strategy and selection criteria

This systematic review adhered to the updated 2020 PRISMA (Preferred Reporting Items for Systematic Reviews and Meta-Analyses) guidelines [10]. Our study was registered in INPLASY platform (number: INPLASY202560016). We included RCTs that compared intensive versus standard blood pressure control for cardiovascular outcomes without restrictions on publication language or status. A comprehensive literature search was conducted across PubMed, Embase, Web of Science, and the Cochrane Library for eligible studies published up to May 2025. Search terms included ‘Blood Pressure Monitors’ [MeSH], ‘Blood Pressure Determination’, ‘Blood Pressure Control’, and related variants (detailed search strategies for each database are provided in Supplementary File 1). We also searched ClinicalTrials.gov (National Institutes of Health, USA) for completed but unpublished trials and manually screened the reference lists of relevant reviews and articles to identify additional eligible studies.

Two reviewers independently conducted the literature search and study selection using a standardized protocol. Discrepancies were resolved through discussion with a third reviewer until a consensus was reached. Studies were included based on the following criteria: (1) Participants: patients diagnosed with hypertension; (2) Intervention: intensive blood pressure control, defined per trial protocol as aiming for a lower systolic/diastolic target (e.g. SBP <120 or <130 mmHg); (3) Comparator: standard blood pressure control, defined per trial protocol as aiming for a higher systolic/diastolic target (e.g. SBP <140 mmHg); (4) Outcomes: reporting at least one major cardiovascular event (MACE), myocardial infarction (MI), stroke, all-cause mortality, or cardiac death; and (5) Study design: RCT. For multiple publications involving the same population, we included either the primary publication or the study reporting the most comprehensive data.

### Data collection and quality assessment

Two reviewers independently extracted relevant data from the included studies using a standardized protocol, resolving disagreements through discussion until consensus was achieved. Extracted data included the first author’s surname or study group name, publication year, sample size, mean age, proportion of male participants, body mass index (BMI), smoking status, diabetes status, history of stroke, history of myocardial infarction, baseline and post-treatment blood pressure levels in both intervention and control groups, blood pressure target, follow-up duration, and reported outcomes.

The methodological quality of each trial was assessed using the Cochrane Risk of Bias (RoB) tool [11]. We evaluated key domains, including random sequence generation, allocation concealment, blinding of participants and personnel, blinding of outcome assessment, incomplete outcome data, selective reporting, and other sources of bias. Quality assessments were performed independently by two reviewers, with disagreements resolved by a third reviewer.

### Statistical analysis

The primary analysis compared intensive versus standard blood pressure management strategies, irrespective of the specific antihypertensive medication regimens used in the individual trials. We calculated the effect of intensive versus standard blood pressure control on cardiovascular outcomes using relative risk (RR) with corresponding 95% confidence intervals (CI). All pooled estimates were derived using a random-effects model [12,13] to account for potential heterogeneity among studies. Heterogeneity was assessed using the *I^2^* statistic and Cochran’s Q test, with significant heterogeneity defined as *I^2^* ≥ 50% or a Q test *P*-value < 0.10 [14,15].

To evaluate the robustness of the findings and explore sources of heterogeneity, we conducted sensitivity analyses by sequentially excluding individual studies and recalculating pooled effect estimates [16]. Moreover, we conducted post hoc subgroup analyses at the study level by categorizing trials based on their aggregate baseline characteristics: sample size (<1000 vs. ≥1000), mean age (<65 vs. ≥65 years), proportion of male participants (<60% vs. ≥60%), BMI (<27.5% vs. ≥27.5 kg/m^2^), current smoking proportion (<20% vs. ≥20%), diabetes status (diabetic patients vs. Non-diabetic patients or both), and follow-up duration (<5 vs. ≥5 years). Differences between subgroups were assessed using tests for interaction, which assuming the data met normal distribution [17].

Publication bias was examined through visual inspection of funnel plot symmetry and formally assessed using Egger’s test and Begg’s test [18,19]. All effect estimates were reported with two-sided *P*-values, and statistical significance was defined as *p* < 0.05. Analyses were conducted using STATA software, version 12.0 (StataCorp LP, College Station, TX, USA).

## Results

### Literature search

**Figure 1.**
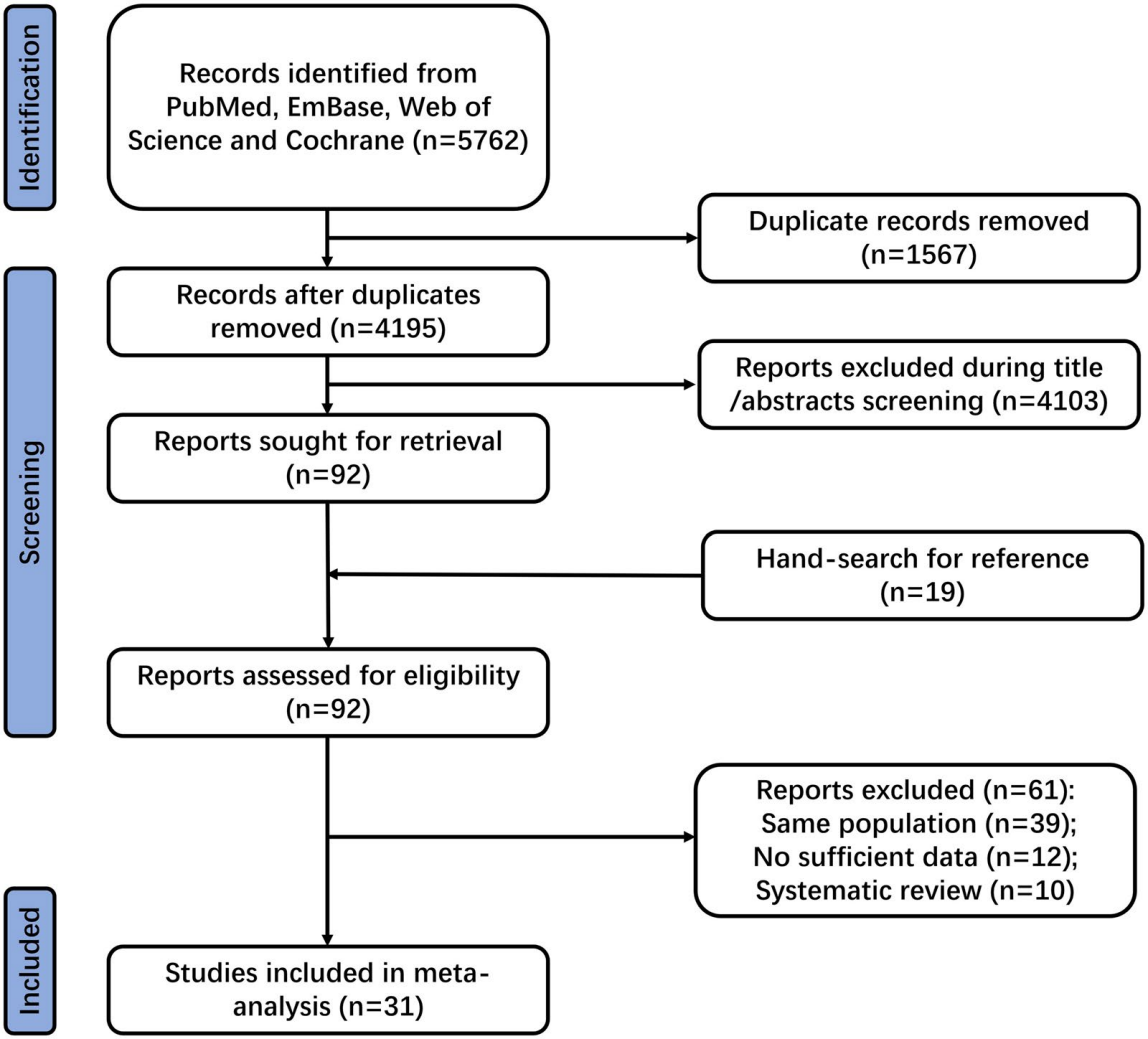
PRISMA flow diagram of the literature search and study selection process. This diagram illustrates the identification, screening, eligibility assessment, and final inclusion of studies in the meta-analysis. The numbers of records identified, excluded, and included at each stage are shown.

A total of 5,762 publications were identified through database searches. After removing 1,567 duplicates, 4,103 records were excluded based on title or abstract screening. The remaining 92 articles underwent full-text review, with 61 excluded for the following reasons: duplicate reports from the same population (*n* = 39), absence of relevant outcomes (*n* = 12), and classification as systematic reviews (*n* = 10). Manual screening of reference lists yielded 19 additional articles, all of which had already been captured by the electronic search. Ultimately, 31 RCTs met the inclusion criteria and were included in the final meta-analysis [20–50]. The study selection process is illustrated in Figure 1.

### Trials characteristics

Baseline characteristics of the included studies and participants are summarized in Table 1. The 31 RCTs enrolled a total of 156,933 participants, with individual sample sizes ranging from 81 to 33,995. Participant ages ranged from 36.6 to 83.6 years; the proportion of male participants varied between 34.5% and 69.4%, and follow-up duration ranged from 1.8 to 19.3 years. The specific blood pressure targets for the intensive and standard control arms in each trial are detailed in Table 1. The methodological quality assessment results are presented in Supplementary File 2 (Table S1) and indicate that most studies were of high quality, with a generally low risk of bias across key domains.

**Table 1.** The baseline characteristics of included studies and involved patients.

Study, year	Sample size, N (I/S)	Age (years)	Male (%)	BMI (kg/m^2^)	Smoking (%)	Diabetes (%)	Stroke (%)	MI (%)	Baseline BP (mmHg) (SBP/DBP)	BP target (I/S, mmHg)	BP after treatment (I, SBP/DBP)	BP after treatment (S, SBP/DBP)	Follow-up (years)
SHEP 1991 [20]	4736 (2365/2371)	71.5	43.2	27.5	12.7	10.1	1.4	4.9	170.3/76.5	> 20 reduction	144.0/67.7	155.1/71.1	4.5
HOT 1998 [21]	12526 (6262/6264)	61.5	53.0	28.4	15.9	8.0	1.2	1.6	170.0/105.0	DBP ≤ 90 vs < 80	139.7/81.1	143.7/85.2	3.8
UKPDS 38 1998 [22]	1148 (758/390)	56.4	55.5	29.6	23.0	100.0	NA	NA	159.3/94.0	<150/85 vs <180/105	144.0/82.0	154.0/87.0	8.4
ABCD 2000 [23]	470 (237/233)	57.9	67.4	31.8	NA	100.0	NA	NA	155.0/98.0	DBP ≤ 75 vs 80–90	132.0/78.0	138.0/86.0	5.3
AASK 2002 [24]	1094 (540/554)	54.6	61.2	NA	NA	NA	NA	NA	150.5/95.5	MAP ≤ 92 vs 102–107	128.0/78.0	141.0/85.0	3.0-6.4
SCOPE 2003 [25]	4937 (2477/2460)	76.4	35.5	27.0	8.7	12.1	3.9	4.5	166.2/90.3	NA	145.2/79.9	148.5/81.6	3.7
FEVER 2005 [26]	9711 (4841/4870)	61.5	61.0	26.3	29.1	12.8	14.8	1.9	154.3/91.2	NA	138.1/82.3	141.6/83.9	3.3
ABCD 2V 2006 [27]	129 (66/63)	56.1	67.5	32.2	10.1	100.0	0.8	2.3	126.0/84.0	DBP ≤ 75 vs 80–90	118.0/75.0	124.0/80.0	1.9
ADVANCE 2007 [28]	11140 (5569/5571)	66.0	57.0	28.0	15.0	100.0	9.0	12.0	145.0/81.0	NA	136.0/73.0	140.0/73.0	4.3
HYVET 2008 [29]	3845 (1933/1912)	83.6	39.5	24.7	6.5	6.8	6.8	3.1	173.0/90.8	NA	143.5/77.9	158.5/84.0	1.8
JATOS 2008 [30]	4418 (2212/2206)	73.6	38.9	23.6	13.5	11.8	4.3	NA	171.6/89.1	NA	135.9/74.8	145.6/78.1	2.0
SANDS 2008 [31]	499 (252/247)	56.0	34.5	33.5	20.4	100.0	NA	NA	130.5/75.0	SBP ≤ 115 vs ≤ 130	117.0/67.0	129.0/73.0	3.0
Cardio-Sis 2009 [32]	1111 (558/553)	67.0	41.0	27.8	21.5	0.0	8.5	NA	163.3/98.6	SBP ≤ 130 vs ≤ 140	131.9/77.4	135.6/78.7	2.0
ACCORD 2010 [33]	4733 (2362/2371)	62.2	52.3	32.1	13.2	100.0	NA	NA	139.2/76.0	SBP ≤ 120 vs ≤ 140	119.3/64.4	133.5/70.5	4.7
VALISH 2010 [34]	3079 (1545/1534)	76.1	37.6	23.5	19.2	13.0	6.6	NA	169.5/81.5	SBP ≤ 140 vs 140–150	133.6/74.8	142.0/76.5	3.1
HOMED-BP 2012 [35]	3518 (1759/1759)	59.6	50.0	24.4	22.0	15.0	NA	NA	154.2/90.2	SBP ≤ 125 vs 125–134	131.6/76.5	132.8/76.9	5.3
Wei 2013 [36]	724 (363/361)	76.6	66.3	23.4	24.9	23.3	6.6	NA	159.5/86.0	SBP ≤ 140 vs ≤ 150	135.7/76.2	149.7/82.1	4.0
SPS3 2013 [37]	3020 (1501/1519)	63.0	63.0	29.1	20.5	36.5	100.0	NA	143.0/78.5	SBP ≤ 130 vs 130–149	127.0/NA	138.0/NA	3.7
Syst-Eur 2014 [38]	4695 (2398/2297)	70.3	33.2	27.0	7.3	10.5	NA	NA	173.8/85.5	NA	149.8/NA	160.3/NA	2.0
HALT-PKD 2014 [39]	558 (274/284)	36.6	50.7	27.2	NA	NA	NA	NA	126.7/80.1	SBP ≤ 110 vs ≤ 120	95.0-110.0/60.0-75.0	120.0-130.0/70.0-80.0	8.0
MDRD 2015 [40]	840 (432/408)	51.7	60.5	NA	NA	5.1	NA	NA	NA	MAP ≤ 92 vs 102–107	125.0/75.0	140.0/90.0	19.3
SPRINT 2015 [41]	9361 (4678/4683)	67.9	64.4	29.8	13.2	NA	NA	NA	139.7/78.1	SBP ≤ 120 vs ≤ 140	121.4/68.7	136.2/76.3	3.3
J-DOIT3 2017 [42]	2540 (1269/1271)	59.0	62.0	24.9	23.4	100.0	NA	NA	133.8/79.7	SBP ≤ 120 vs ≤ 130	124.9/71.3	128.2/72.9	8.5
ADDITION-L 2019 [43]	336 (144/192)	59.5	57.8	30.6	11.6	100.0	NA	NA	147.2/89.0	SBP ≤ 130 vs ≤ 140	126.9/73.7	139.2/80.9	5.0
INFINITY 2019 [44]	199 (99/100)	80.6	45.7	27.9	1.0	15.1	NA	NA	150.9/75.6	SBP ≤ 130 vs ≤ 145	127.7/64.6	144.0/72.3	3.0
RESPECT 2019 [45]	1263 (633/630)	67.2	69.4	23.8	NA	23.4	100.0	2.5	145.4/83.6	SBP ≤ 120 vs ≤ 140	126.7/74.4	133.2/77.7	3.9
PRESERVE 2021 [46]	81 (42/39)	68.8	59.3	NA	13.6	2.5	NA	4.9	148.6/NA	SBP ≤ 125 vs 130–140	127.0/NA	140.0/NA	2.0
STEP 2021 [47]	8511 (4243/4268)	66.3	46.5	25.6	NA	19.1	NA	NA	146.0/82.5	SBP ≤ 130 vs ≤ 150	126.7/76.4	135.9/79.2	3.3
CRHCP 2023 [48]	33995 (17407/16588)	63.0	38.7	25.9	21.7	8.9	NA	NA	156.2/87.7	SBP ≤ 130 vs ≤ 140	126.1/73.1	147.6/82.3	3.0
ESPRIT 2024 [49]	11255 (5624/5631)	64.6	58.7	26.3	31.2	38.7	26.8	NA	146.9/82.9	SBP ≤ 120 vs ≤ 140	119.1/NA	134.8/NA	3.4
BPROAD 2025 [50]	12821 (6414/6407)	63.8	54.7	26.7	25.1	100.0	NA	NA	140.2/76.3	SBP ≤ 120 vs ≤ 140	121.6/NA	133.2/NA	4.2

BMI: body mass index; BP: blood pressure; DBP: diastolic BP; I: intensive BP control; MAP: mean artery pressure; MI: myocardial infarction; NA: not available; S: standard BP control; SBP: systolic BP.

### Major cardiovascular events

**Figure 2.**
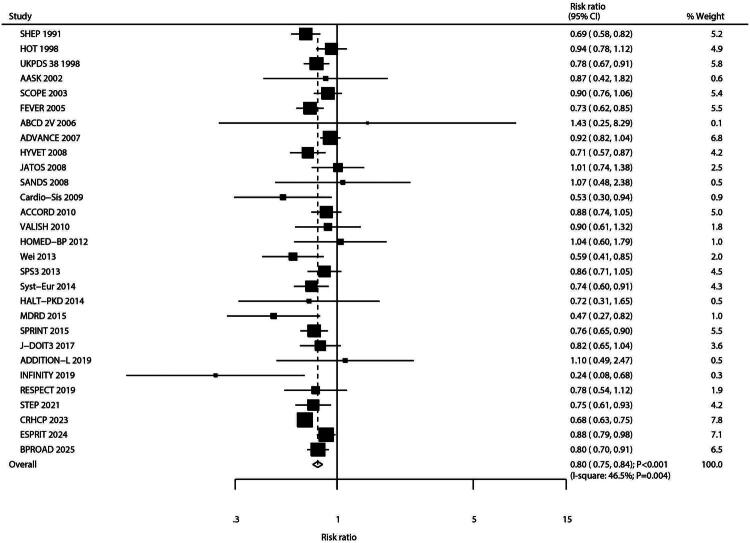
Forest plot for the association between intensive versus standard blood pressure control and major cardiovascular events (MACEs). This random-effects meta-analysis of randomized controlled trials is presented as a forest plot. Each horizontal line represents the 95% confidence interval (CI) for an individual study, and the square represents its point estimate (relative risk, RR). The area of each square is proportional to the study’s weight in the meta-analysis. The dashed vertical line indicates the line of overall effect (pooled RR). The diamond at the bottom represents the overall pooled RR with its 95% CI. Heterogeneity among studies was quantified as I-square = 46.5%.

Twenty-nine studies reported data on major adverse cardiovascular events (MACEs). Intensive blood pressure control was associated with a significant reduction in MACE risk compared to standard control (RR: 0.80; 95% CI: 0.75–0.84; *p* < 0.001; Figure 2). Moderate heterogeneity was observed across studies (*I^2^* = 46.5%; *p* = 0.004). Sensitivity analysis confirmed that the result was robust and not unduly influenced by any single study (Figure S1). Subgroup analysis showed a consistent reduction in MACE risk across all subgroups, although the effect size was attenuated among patients with diabetes (Table 2). Funnel plot inspection and statistical tests (Egger’s test *p* = 0.887; Begg’s test *p* = 0.511; Figure S2) revealed no significant evidence of publication bias.

**Table 2.** Subgroup analyses for the association between intensive vs. standard blood pressure control and cardiovascular outcomes.

Outcomes	Factors	Subgroups	RR and 95%CI	*P* value	*I^2^* (%)	Q statistic	Interaction *P* test^
Major cardiovascular events	Sample size	≥1000	0.80 (0.76–0.85)	<0.001	47.1	0.008	0.054
		<1000	0.64 (0.46–0.90)	0.010	32.5	0.180	
	Mean age (years)	≥65.0	0.78 (0.71–0.85)	<0.001	52.3	0.014	0.712
		<65.0	0.81 (0.75–0.87)	<0.001	44.5	0.029	
	Male (%)	≥60.0	0.76 (0.70–0.83)	<0.001	0.0	0.480	0.282
		<60.0	0.81 (0.75–0.87)	<0.001	56.4	0.001	
	BMI (kg/m^2^)	≥27.5	0.82 (0.75–0.90)	<0.001	46.1	0.040	0.067
		<27.5	0.78 (0.73–0.84)	<0.001	43.4	0.037	
	Smoking (%)	≥20.0	0.78 (0.71–0.84)	<0.001	51.5	0.024	0.054
		<20.0	0.83 (0.76–0.90)	<0.001	47.8	0.028	
	DM (%)	DM	0.85 (0.80–0.91)	<0.001	0.0	0.630	0.014
		Non-DM or both	0.77 (0.72–0.83)	<0.001	51.2	0.004	
	Follow-up (years)	≥5.0	0.78 (0.68–0.88)	<0.001	2.8	0.391	0.766
		<5.0	0.80 (0.75–0.85)	<0.001	52.2	0.002	
Myocardial infarction	Sample size	≥1000	0.83 (0.76–0.91)	<0.001	0.0	0.950	1.000
		<1000	0.51 (0.07–3.88)	0.518	52.1	0.149	
	Mean age (years)	≥65.0	0.85 (0.73–0.99)	0.038	0.0	0.723	0.721
		<65.0	0.82 (0.73–0.91)	<0.001	0.0	0.904	
	Male (%)	≥60.0	0.85 (0.69–1.05)	0.135	0.0	0.756	0.814
		<60.0	0.83 (0.75–0.91)	<0.001	0.0	0.844	
	BMI (kg/m^2^)	≥27.5	0.80 (0.71–0.90)	<0.001	0.0	0.790	0.410
		<27.5	0.86 (0.76–0.99)	0.033	0.0	0.866	
	Smoking (%)	≥20.0	0.82 (0.72–0.94)	0.004	0.0	0.940	0.826
		<20.0	0.83 (0.74–0.94)	0.003	0.0	0.613	
	DM (%)	DM	0.83 (0.71–0.96)	0.015	0.0	0.693	0.968
		Non-DM or both	0.83 (0.74–0.93)	0.001	0.0	0.862	
	Follow-up (years)	≥5.0	0.77 (0.57–1.02)	0.071	2.4	0.311	0.563
		<5.0	0.84 (0.76–0.92)	<0.001	0.0	0.929	
Stroke	Sample size	≥1000	0.76 (0.70–0.83)	<0.001	45.0	0.014	0.340
		<1000	0.61 (0.40–0.95)	0.027	0.0	0.955	
	Mean age (years)	≥65.0	0.77 (0.69–0.86)	<0.001	18.0	0.257	0.328
		<65.0	0.75 (0.68–0.84)	<0.001	47.9	0.032	
	Male (%)	≥60.0	0.73 (0.63–0.85)	<0.001	18.3	0.290	0.704
		<60.0	0.77 (0.70–0.84)	<0.001	40.8	0.034	
	BMI (kg/m^2^)	≥27.5	0.78 (0.67–0.92)	0.002	46.3	0.061	0.178
		<27.5	0.74 (0.68–0.81)	<0.001	27.3	0.155	
	Smoking (%)	≥20.0	0.74 (0.66–0.83)	<0.001	50.4	0.034	0.212
		<20.0	0.79 (0.70–0.90)	<0.001	32.2	0.133	
	DM (%)	DM	0.70 (0.55–0.90)	0.006	72.2	0.006	0.187
		Non-DM or both	0.75 (0.70–0.80)	<0.001	8.2	0.352	
	Follow-up (years)	≥5.0	0.52 (0.37–0.73)	<0.001	0.0	0.718	0.029
		<5.0	0.77 (0.72–0.83)	<0.001	34.0	0.061	
All-cause mortality	Sample size	≥1000	0.89 (0.85–0.93)	<0.001	2.0	0.433	0.011
		<1000	0.71 (0.60–0.84)	<0.001	0.0	0.625	
	Mean age (years)	≥65.0	0.87 (0.82–0.93)	<0.001	0.0	0.832	1.000
		<65.0	0.88 (0.80–0.96)	0.006	39.8	0.051	
	Male (%)	≥60.0	0.81 (0.71–0.92)	0.001	29.1	0.186	0.038
		<60.0	0.89 (0.85–0.94)	<0.001	0.0	0.613	
	BMI (kg/m^2^)	≥27.5	0.90 (0.81–1.00)	0.048	38.3	0.086	0.107
		<27.5	0.87 (0.82–0.93)	<0.001	0.0	0.713	
	Smoking (%)	≥20.0	0.86 (0.80–0.92)	<0.001	0.0	0.536	0.148
		<20.0	0.90 (0.84–0.97)	0.005	16.5	0.273	
	DM (%)	DM	0.91 (0.80–1.12)	0.100	24.7	0.232	0.408
		Non-DM or both	0.86 (0.82–0.92)	<0.001	10.2	0.325	
	Follow-up (years)	≥5.0	0.78 (0.63–0.97)	0.022	34.9	0.175	0.064
		<5.0	0.89 (0.85–0.93)	<0.001	0.0	0.512	
Cardiac death	Sample size	≥1000	0.80 (0.74–0.87)	<0.001	9.3	0.345	0.030
		<1000	0.50 (0.32–0.76)	0.001	0.0	0.854	
	Mean age (years)	≥65.0	0.80 (0.72–0.88)	<0.001	6.6	0.381	0.745
		<65.0	0.79 (0.69–0.92)	0.002	30.4	0.166	
	Male (%)	≥60.0	0.67 (0.55–0.81)	<0.001	3.3	0.388	0.059
		<60.0	0.82 (0.75–0.89)	<0.001	6.5	0.380	
	BMI (kg/m^2^)	≥27.5	0.85 (0.74–0.99)	0.038	26.9	0.214	0.265
		<27.5	0.75 (0.68–0.83)	<0.001	4.1	0.403	
	Smoking (%)	≥20.0	0.69 (0.62–0.78)	<0.001	0.0	0.676	0.018
		<20.0	0.86 (0.78–0.95)	0.003	8.1	0.368	
	DM (%)	DM	0.83 (0.72–0.96)	0.013	0.0	0.425	0.421
		Non-DM or both	0.78 (0.70–0.86)	<0.001	22.0	0.204	
	Follow-up (years)	≥5.0	–	–	–	–	–
		<5.0	0.79 (0.73–0.86)	<0.001	16.2	0.252	

^Interaction *P* test values were derived comparing subgroup effect estimates.

BMI: body mass index; CI: confidence interval; DM: diabetes mellitus; RR: relative risk.

### Myocardial infarction

**Figure 3.**
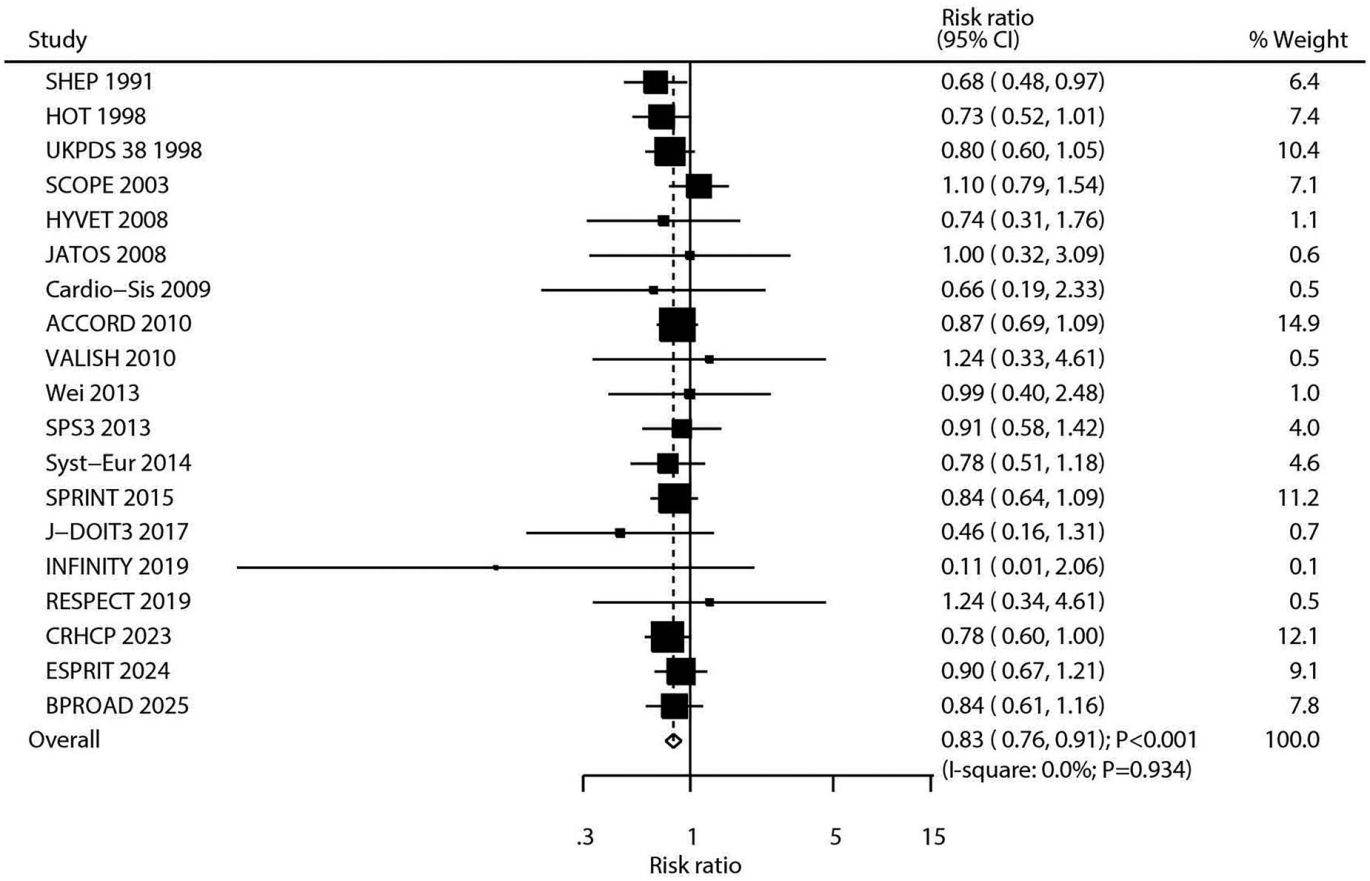
Forest plot for the association between intensive versus standard blood pressure control and myocardial infarction (MI). This random-effects meta-analysis of randomized controlled trials is presented as a forest plot. Each horizontal line represents the 95% confidence interval (CI) for an individual study, and the square represents its point estimate (relative risk, RR). The area of each square is proportional to the study’s weight in the meta-analysis. The dashed vertical line indicates the line of overall effect (pooled RR). The diamond at the bottom represents the overall pooled RR with its 95% CI. Heterogeneity among studies was quantified as I-square = 0.0%.

Nineteen studies reported data on MI outcomes. Intensive blood pressure control significantly reduced the risk of MI compared to standard control (RR: 0.83; 95% CI: 0.76–0.91; *p* < 0.001; Figure 3), with no significant heterogeneity observed (*I^2^* = 0.0%; *p* = 0.934). Sensitivity analysis confirmed the stability of the finding (Figure S3). Subgroup analyses indicated that the protective effect of intensive control was consistent across most subgroups; however, no significant risk reduction was observed in studies with a sample size < 1,000, male proportion ≥ 60.0%, or follow-up duration ≥ 5.0 years (Table 2). Evaluation for publication bias using funnel plots and formal statistical tests showed no significant bias (Egger’s test *p* = 0.535; Begg’s test *p* = 0.889; Figure S4).

### Stroke

**Figure 4.**
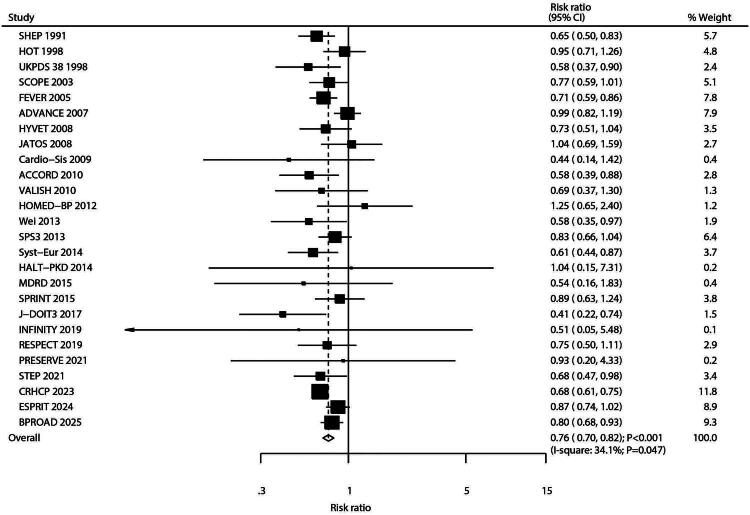
Forest plot for the association between intensive versus standard blood pressure control and stroke. This random-effects meta-analysis of randomized controlled trials is presented as a forest plot. Each horizontal line represents the 95% confidence interval (CI) for an individual study, and the square represents its point estimate (relative risk, RR). The area of each square is proportional to the study’s weight in the meta-analysis. The dashed vertical line indicates the line of overall effect (pooled RR). The diamond at the bottom represents the overall pooled RR with its 95% CI. Heterogeneity among studies was quantified as I-square = 34.1%.

Twenty-six studies reported comparisons of stroke risk between intensive and standard blood pressure control. Intensive control was associated with a significant reduction in stroke risk (RR: 0.76; 95% CI: 0.70–0.82; *p* < 0.001; Figure 4), with moderate heterogeneity observed across studies (*I^2^* = 34.1%; *p* = 0.047). Sensitivity analysis confirmed the robustness of this finding, indicating that no single study substantially influenced the overall result (Figure S5). Subgroup analyses consistently demonstrated significant stroke risk reduction with intensive control across all subgroups; however, the magnitude of reduction was greater in studies with a follow-up duration ≥ 5.0 years (Table 2). Funnel plot symmetry and statistical tests (Egger’s test *p* = 0.730; Begg’s test *p* = 0.537; Figure S6) showed no significant evidence of publication bias for the stroke outcome.

### All-cause mortality

Twenty-nine studies reported data on all-cause mortality, comparing intensive versus standard blood pressure control. Intensive control significantly reduced all-cause mortality risk (RR: 0.87; 95% CI: 0.83–0.92; *p* < 0.001; Figure S7), with low heterogeneity observed among studies (*I^2^* = 13.2%; *p* = 0.264). Sensitivity analysis affirmed the robustness of this finding, indicating that the result was not substantially influenced by any single study (Figure S8). Subgroup analysis revealed significant mortality reductions across most subgroups; however, no significant benefit was observed among patients with diabetes (Table 2). Evaluation of publication bias using funnel plot inspection and statistical tests (Egger’s test *p* = 0.570; Begg’s test *p* = 0.442; Figure S9) indicated no significant bias for this outcome.

### Cardiac death

Twenty studies reported data comparing the risk of cardiac death between intensive and standard blood pressure control. Intensive control significantly reduced the risk of cardiac death (RR: 0.79; 95% CI: 0.73–0.86; *p* < 0.001; Figure S10), with low heterogeneity observed (*I^2^* = 16.2%; *p* = 0.252). Sensitivity analysis confirmed the stability of this result (Figure S11). Subgroup analyses showed significant reductions in cardiac death risk across all subgroups; notably, the magnitude of reduction appeared to be influenced by sample size and smoker status (Table 2). Funnel plot inspection and statistical tests (Egger’s test *p* = 0.476; Begg’s test *p* = 0.417; Figure S12) showed no significant evidence of publication bias for the cardiac death outcome.

## Discussion

This meta-analysis of 31 RCTs, encompassing nearly 160,000 patients with hypertension, demonstrates that intensive blood pressure control significantly improves cardiovascular outcomes and reduces all-cause mortality compared to conventional management. These findings provide pivotal evidence in the ongoing debate over optimal blood pressure targets and have substantial implications for clinical practice.

The analysis revealed a 20% relative reduction in MACEs with intensive control, with stroke prevention showing the greatest benefit (24% risk reduction). This underscores the particular sensitivity of the cerebrovascular system to blood pressure fluctuations. Additionally, the observed 13% reduction in all-cause mortality and 21% decrease in cardiac death validate the clinical value of intensive blood pressure management through improvements in both overall survival and cardiac-specific outcomes.

While consistent with previous studies supporting intensive blood pressure lowering, this meta-analysis addresses limitations in prior research, particularly incomplete coverage of the literature [51]. Earlier investigations often focused on specific subpopulations, such as patients with diabetes [52], chronic kidney disease [53], or adults over 60 years of age [54], which limited the generalizability of their findings. Although safety was not a primary outcome in our analysis, clinicians aiming for lower blood pressure targets should closely monitor treatment tolerance and potential adverse events to ensure an optimal balance between benefit and risk.

From a mechanistic standpoint, intensive blood pressure control more effectively reduces vascular wall stress, slows atherosclerotic progression, and lowers the risk of plaque rupture and thrombosis, thereby decreasing the incidence of myocardial infarction and stroke [55]. Additionally, rigorous blood pressure control reduces cardiac afterload and myocardial oxygen demand, which helps preserve cardiac function and lowers the risk of cardiac and all-cause mortality [56]. These benefits are consistent with the known pathophysiology of hypertension, particularly the role of excessive activation of the renin-angiotensin-aldosterone system. More intensive suppression of this system may underlie the enhanced cardiovascular protection associated with aggressive blood pressure management [57].

Despite clear overall benefits, significant heterogeneity in treatment responses was observed across specific population subgroups. Notably, patients with diabetes showed no significant improvement in all-cause mortality and experienced smaller reductions in MACEs compared to non-diabetic individuals. This may be attributed to diabetes-specific pathophysiological mechanisms, such as hyperglycemia-induced endothelial dysfunction, increased oxidative stress, and autonomic dysregulation, which may blunt the cardiovascular benefits of intensive blood pressure lowering [58,59].

In addition, our analysis revealed that the reduction in MI risk did not reach statistical significance in subgroups with higher proportions of male participants (≥60%), longer follow-up durations (≥5 years), or smaller sample sizes (<1,000 patients). While this may reflect a ‘diminishing returns’ effect where the incremental benefit of intensive control attenuates over very long periods, it could also be influenced by competing risks in aging populations, changes in concomitant therapies over time, or the play of chance given the smaller number of trials and events in this specific subgroup. This observation warrants further investigation in long-term follow-up studies.

Importantly, the observed benefit of intensive control on cardiac death was notably attenuated in populations with higher smoking prevalence. This suggests that nicotine-induced direct cardiovascular damage may partially counteract the protective effects of antihypertensive therapy, emphasizing the critical need to incorporate smoking cessation strategies into comprehensive blood pressure management protocols [60]. Nonetheless, the limited number of studies in certain subgroups may reduce the statistical power and reliability of these subgroup-specific findings.

This study has several limitations. Firstly, although it includes a large number of trials and participants, differences in the specific blood pressure targets used to define intensive and standard control, blood pressure measurement protocols, treatment regimens, and baseline patient characteristics across studies may have contributed to heterogeneity in some outcomes. Secondly, we did not perform a stratified analysis based on specific antihypertensive drug classes used in the intensive versus standard strategies. Differences in medication regimens could contribute to heterogeneity and represent an area for future research. Thirdly, we did not assess the impact of prolonged intensive control on patient-reported outcomes such as quality of life. Fourthly, while the focus of this meta-analysis was on cardiovascular efficacy, we did not systematically analyze safety outcomes such as symptomatic hypotension, electrolyte disturbances, or acute kidney injury. The balance between the cardiovascular benefits demonstrated here and the potential for increased adverse events with intensive control is a critical consideration for clinical practice and should be a focus of future patient-level analyses. Finally, because this meta-analysis relied on aggregated data from published sources, it may be subject to publication bias and lacks the granularity that individual patient data analyses can provide. The exclusion of unpublished or grey literature could also limit the completeness of the evidence base.

## Conclusion

In conclusion, this meta-analysis provides strong evidence that intensive blood pressure control significantly reduces cardiovascular risk, supporting the adoption of lower systolic targets (e.g. <130 mmHg) in clinical practice for most hypertensive patients. To implement this strategy effectively, intensive monitoring is paramount. This should include regular clinic blood pressure checks, consideration of home blood pressure monitoring to assess adherence and response, and vigilance for adverse effects like orthostatic hypotension and electrolyte imbalance, particularly during treatment titration.

Future studies are needed to: (1) identify optimal patient selection criteria using precision medicine approaches to maximize benefit and minimize harm from intensive control; (2) determine the comparative effectiveness and safety of specific antihypertensive drug classes within an intensive strategy framework; (3) evaluate the long-term impact on patient-centered outcomes such as quality of life, cognitive function, and cost-effectiveness; and (4) investigate the best protocols for monitoring and sustaining intensive control in diverse real-world settings, including low-resource environments.

## Supplementary Material

Supplementary_file_1 new.docx

Supplementary_file_2 clean file.docx

Supplementary figures.docx

PRISMA_2020_checklist.docx

## Data Availability

Data will be made available on reasonable request from our corresponding author for current study.
