# Supplementary material for: Effect of intensive versus standard blood pressure control on cardiovascular outcomes: a meta-analysis of randomized controlled trials
Source: Ann Med. 2026 Apr 30;58(1):2662627. doi: 10.1080/07853890.2026.2662627 (PMC13134750; doi:10.1080/07853890.2026.2662627)
Supplement: Supplementary_file_1 new.docx [file IANN_A_2662627_SM6935.docx]

**Supplementary File 1. Search strategy for PubMed, EmBase, Web of Science, and Cochrane Library.**

**Search strategy:**

**PubMed:**

#1: “Blood Pressure Monitors” [MeSH] OR “Monitors, Blood Pressure” OR “Blood Pressure Monitor” OR “Blood Pressure Determination” OR “Blood Pressure Control” OR “Control Blood Pressure”

#2: (randomized controlled trial[Publication Type] OR randomized[TIAB] OR randomised[TIAB] OR placebo[TIAB])) NOT (Review[Publication Type]) NOT (meta-analysis[Publication Type]) NOT (Comment[Publication Type]) NOT (Letter[Publication Type])

#3: #1 AND #2

**EmBase:**

#1: 'Blood pressure monitoring'/exp OR ' Blood pressure regulation':ab,ti OR ' Blood pressure determination ':ab,ti OR ' Blood pressure control ':ab,ti OR ' Control blood p`ressure ':ab,ti

#2: 'randomized controlled trial'/exp NOT review:it

#3: #1 AND #2

**Web of Science:**

(“Blood Pressure Monitors” OR “Monitors, Blood Pressure” OR “Blood Pressure Monitor” OR “Blood Pressure Determination” OR “Blood Pressure Control” OR “Control Blood Pressure”) AND ((randomized controlled trial PT OR randomized TI OR randomized AB OR randomised TI OR randomised AB OR placebo TI OR placebo AB) NOT Review PT NOT meta-analysis PT NOT Comment PT NOT Letter PT)

**Cochrane Library:**

(“Blood Pressure Monitors:ME” OR “Blood Pressure Monitors” OR “Monitors, Blood Pressure” OR “Blood Pressure Monitor” OR “Blood Pressure Determination” OR “Blood Pressure Control” OR “Control Blood Pressure”) AND ((randomized controlled trial PT OR randomized TI OR randomized AB OR randomised TI OR randomised AB OR placebo TI OR placebo AB) NOT Review PT NOT meta-analysis PT NOT Comment PT NOT Letter PT)
