# Supplementary material for: Effect of intensive versus standard blood pressure control on cardiovascular outcomes: a meta-analysis of randomized controlled trials
Source: Ann Med. 2026 Apr 30;58(1):2662627. doi: 10.1080/07853890.2026.2662627 (PMC13134750; doi:10.1080/07853890.2026.2662627)
Supplement: Supplementary_file_2 clean file.docx [file IANN_A_2662627_SM6934.docx]

Supplementary File 2. Risk of bias assessment for included randomized controlled trials.

Table S1. Risk of bias assessment for included randomized controlled trials.

| Study | Random sequence generation | Allocation concealment | Blinding of participants and personnel | Blinding of outcome assessment | Incomplete outcome data | Selective reporting | Other bias |
| --- | --- | --- | --- | --- | --- | --- | --- |
| SHEP 1991 | Low | Low | Low | Low | Low | Low | Unclear |
| HOT 1998 | Low | Low | Low | Low | Low | Low | Unclear |
| UKPDS 38 1998 | Low | Low | Low | Low | Low | Low | Unclear |
| ABCD 2000 | Low | Low | Low | Low | Low | Low | Unclear |
| AASK 2002 | Low | Low | Low | Low | Low | Low | Unclear |
| SCOPE 2003 | Low | Low | Low | Low | Low | Low | Low |
| FEVER 2005 | Low | Low | Low | Low | Low | Low | Unclear |
| ABCD 2V 2006 | Low | Low | Low | Low | Low | Low | Unclear |
| ADVANCE 2007 | Low | Low | Low | Low | Low | Low | Low |
| HYVET 2008 | Low | Low | Low | Low | Low | Low | Unclear |
| JATOS 2008 | Low | Low | Low | Low | Low | Low | Low |
| SANDS 2008 | Low | Low | Low | Low | Low | Low | Low |
| Cardio-Sis 2009 | Low | Low | Low | Low | Low | Low | Low |
| ACCORD 2010 | Low | Low | Low | Low | Low | Low | Low |
| VALISH 2010 | Low | Low | Low | Low | Low | Low | Unclear |
| HOMED-BP 2012 | Low | Low | Low | Low | Low | Low | Unclear |
| Wei 2013 | Low | Low | Low | Low | Low | Low | Unclear |
| SPS3 2013 | Low | Low | Low | Low | Low | Low | Low |
| Syst-Eur 2014 | Low | Low | Low | Low | Low | Low | Unclear |
| HALT-PKD 2014 | Low | Low | Low | Low | Low | Low | Low |
| MDRD 2015 | Low | Low | Low | Low | Low | Low | Unclear |
| SPRINT 2015 | Low | Low | Low | Low | Low | Low | Low |
| J-DOIT3 2017 | Low | Low | Low | Low | Low | Low | Low |
| ADDITION-L 2019 | Low | Low | Low | Low | Low | Low | Unclear |
| INFINITY 2019 | Low | Low | Low | Low | Low | Low | Unclear |
| RESPECT 2019 | Low | Low | Low | Low | Low | Low | Low |
| PRESERVE 2021 | Low | Low | Unclear | Low | Low | Low | Unclear |
| STEP 2021 | Low | Low | Low | Low | Low | Low | Low |
| CRHCP 2023 | Low | Low | Low | Low | Low | Low | Low |
| ESPRIT 2024 | Low | Low | Low | Low | Low | Low | Low |
| BPROAD 2025 | Low | Low | Low | Low | Low | Low | Low |

Footnote: The classification ('Low', 'High', 'Unclear') refers to the risk of bias for each domain according to the Cochrane Risk of Bias Tool criteria. A judgment of 'Low' indicates a low risk of methodological bias for that domain.
