# Supplementary material for: Effect of intensive versus standard blood pressure control on cardiovascular outcomes: a meta-analysis of randomized controlled trials
Source: Ann Med. 2026 Apr 30;58(1):2662627. doi: 10.1080/07853890.2026.2662627 (PMC13134750; doi:10.1080/07853890.2026.2662627)
Supplement: Supplementary figures.docx [file IANN_A_2662627_SM6933.docx]

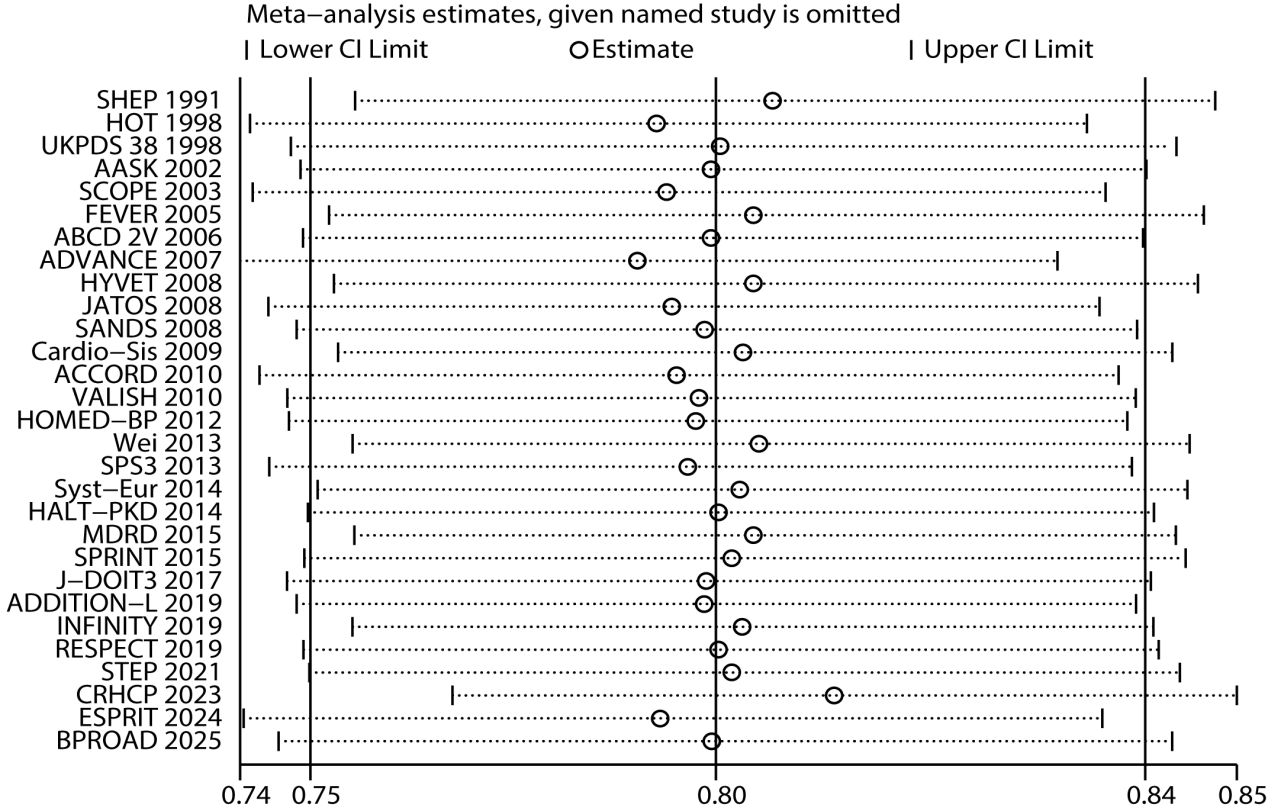


Figure S1. Intensive versus standard blood pressure control on the risk of major cardiovascular events


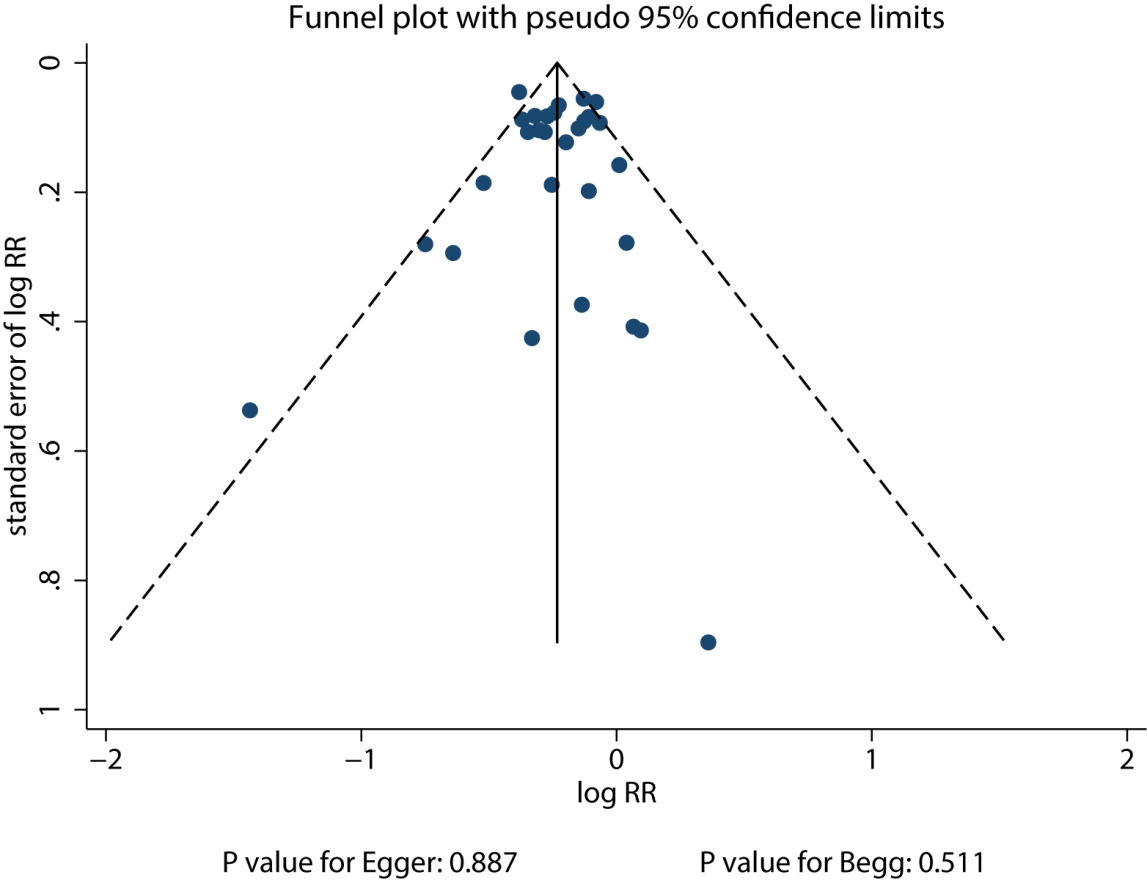


Figure S2. Funnel plot for major cardiovascular events


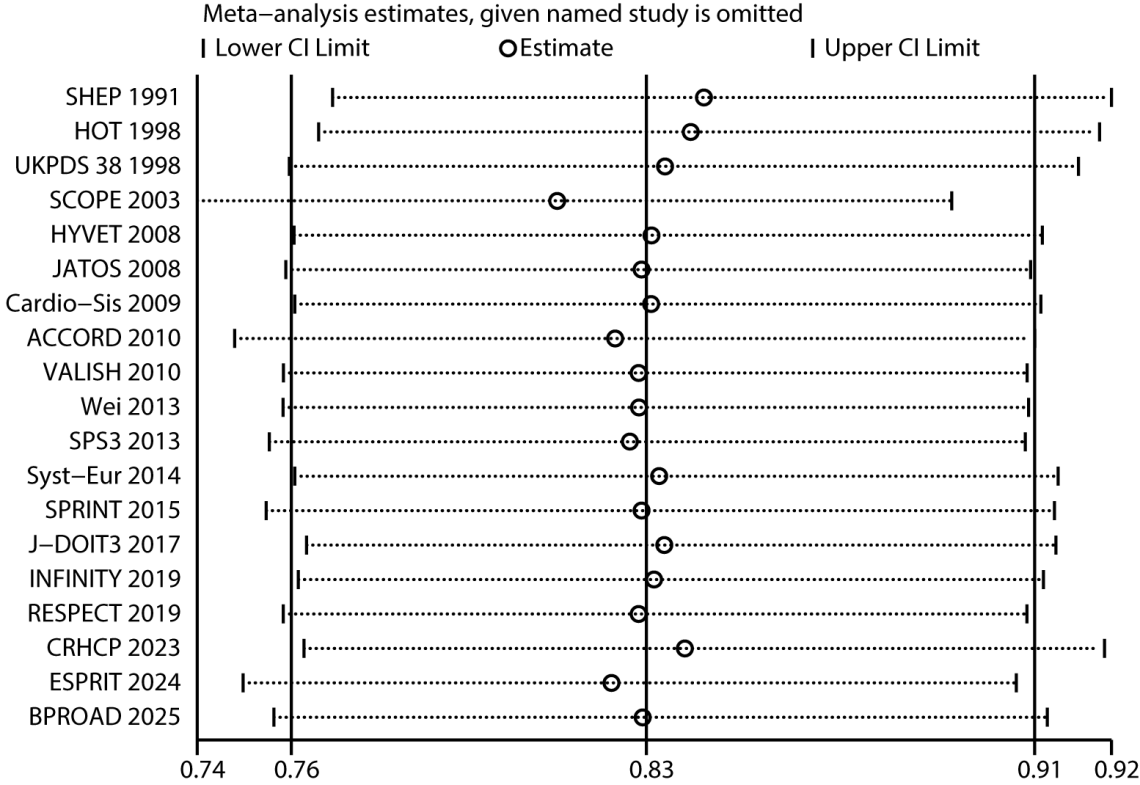


Figure S3. Intensive versus standard blood pressure control on the risk of myocardial infarction


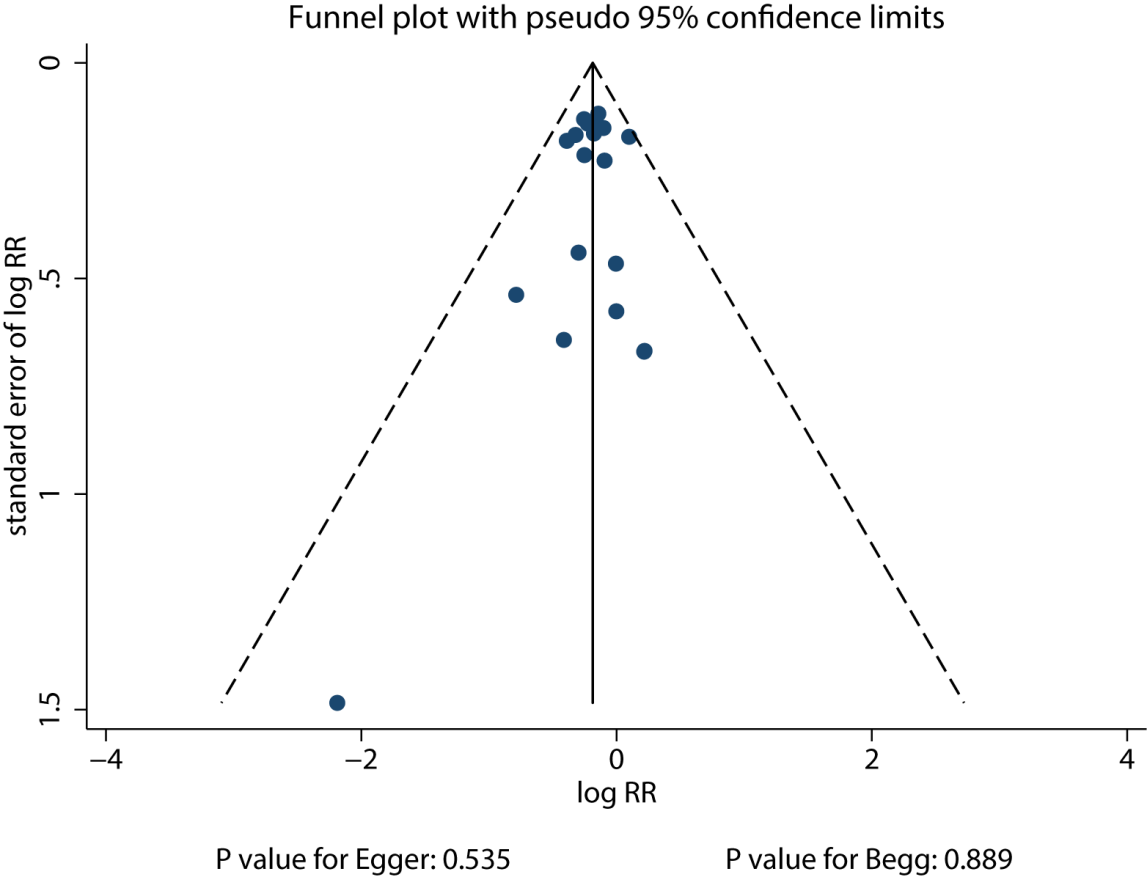


Figure S4. Funnel plot for myocardial infarction


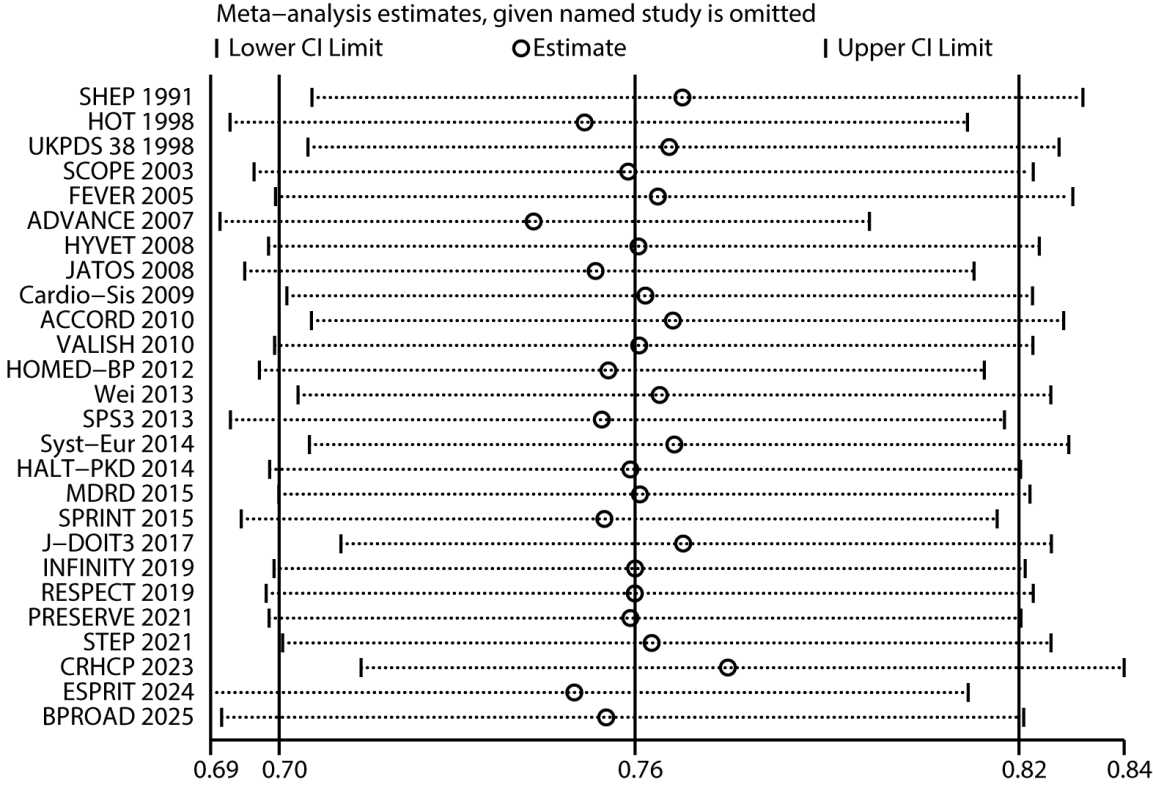


Figure S5. Intensive versus standard blood pressure control on the risk of stroke


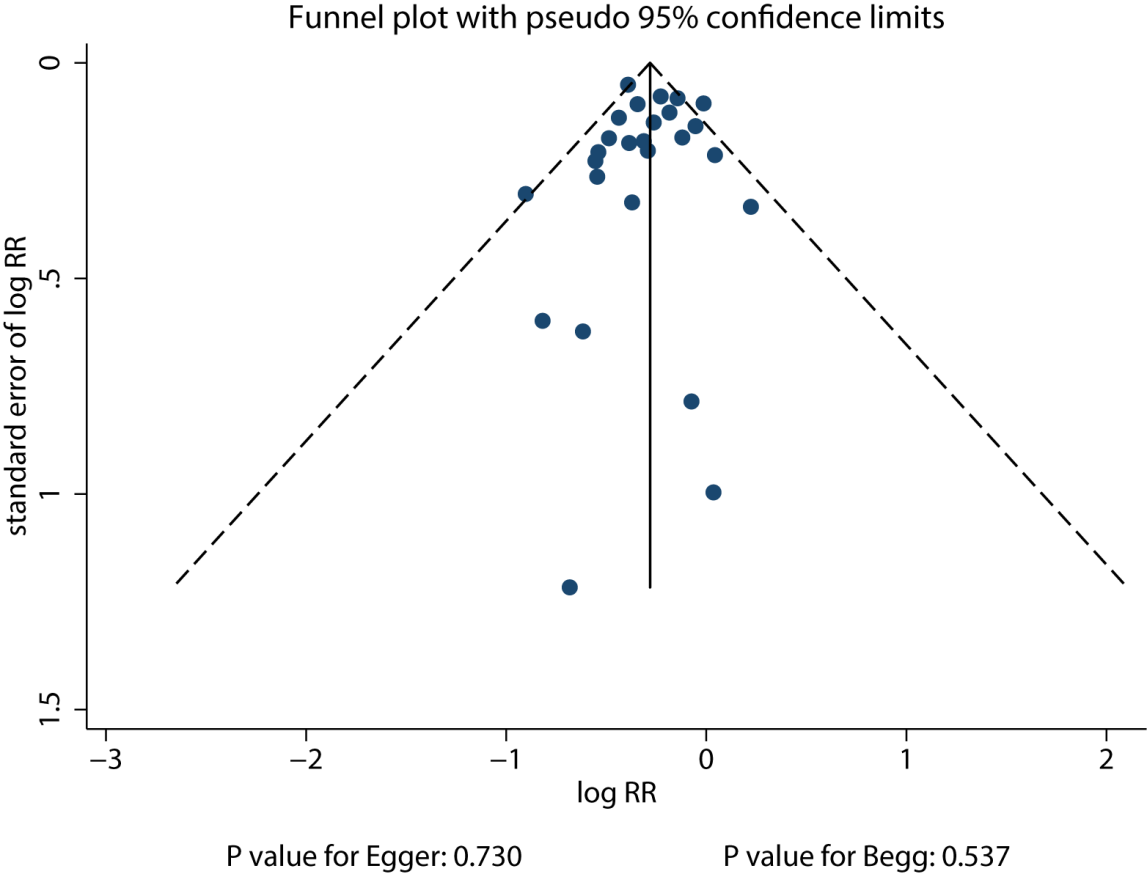


Figure S6. Funnel plot for stroke


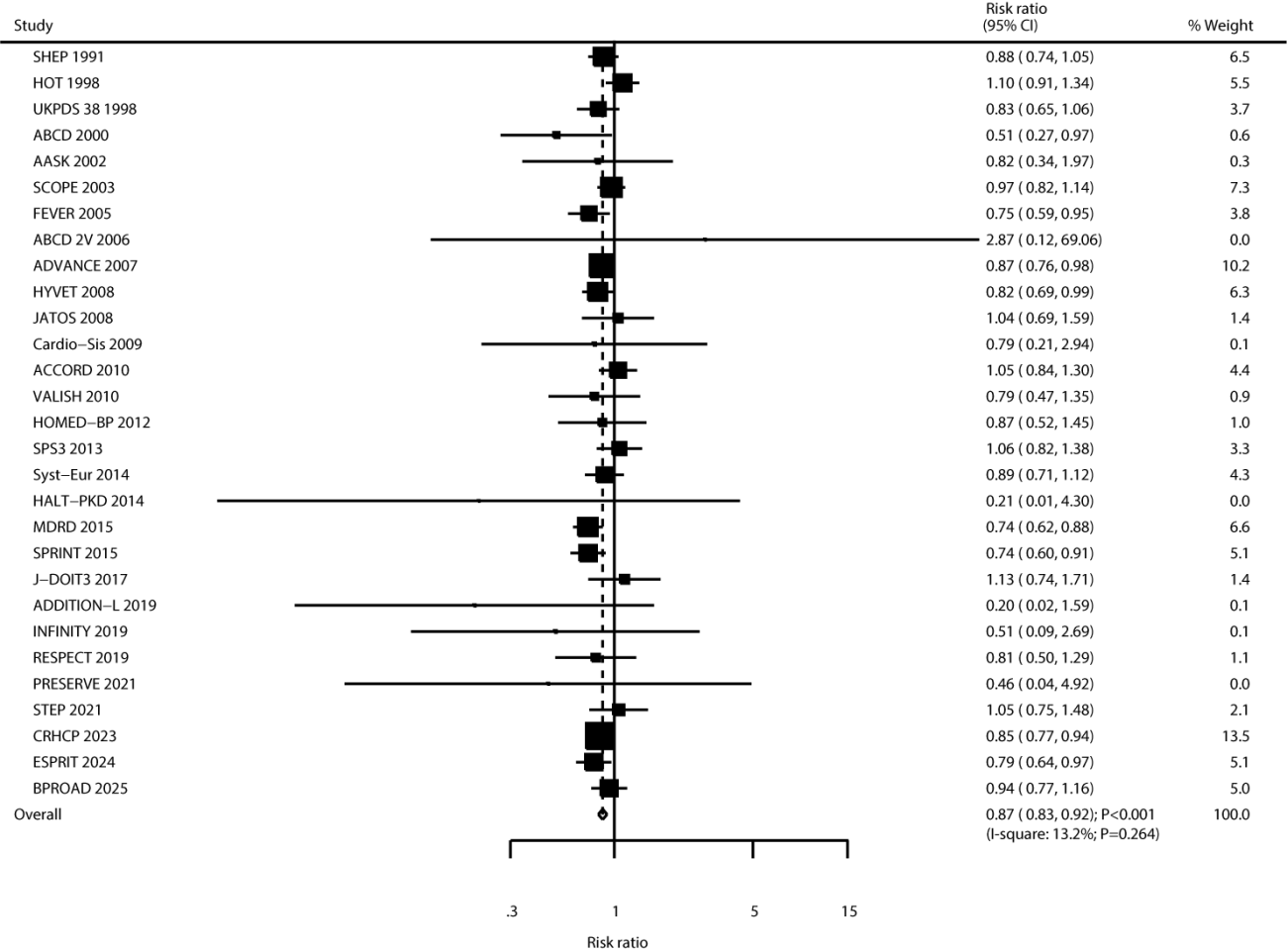


**Figure S7. Forest plot for the association between intensive versus standard blood pressure control and all-cause mortality.** This random-effects meta-analysis of randomized controlled trials is presented as a forest plot. Each horizontal line represents the 95% confidence interval (CI) for an individual study, and the square represents its point estimate (relative risk, RR). The area of each square is proportional to the study's weight in the meta-analysis. The dashed vertical line indicates the line of overall effect (pooled RR). The diamond at the bottom represents the overall pooled RR with its 95% CI. Heterogeneity among studies was quantified as I-square = 13.2%.


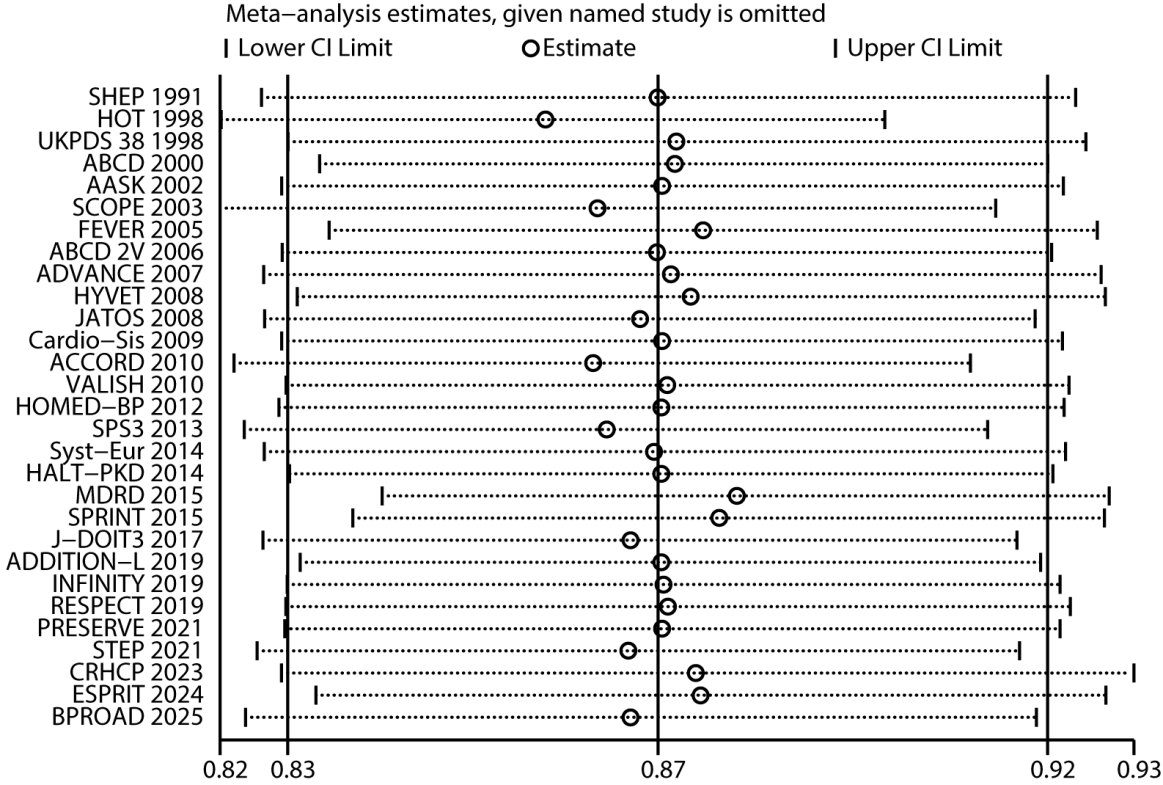


Figure S8. Intensive versus standard blood pressure control on the risk of all-cause mortality


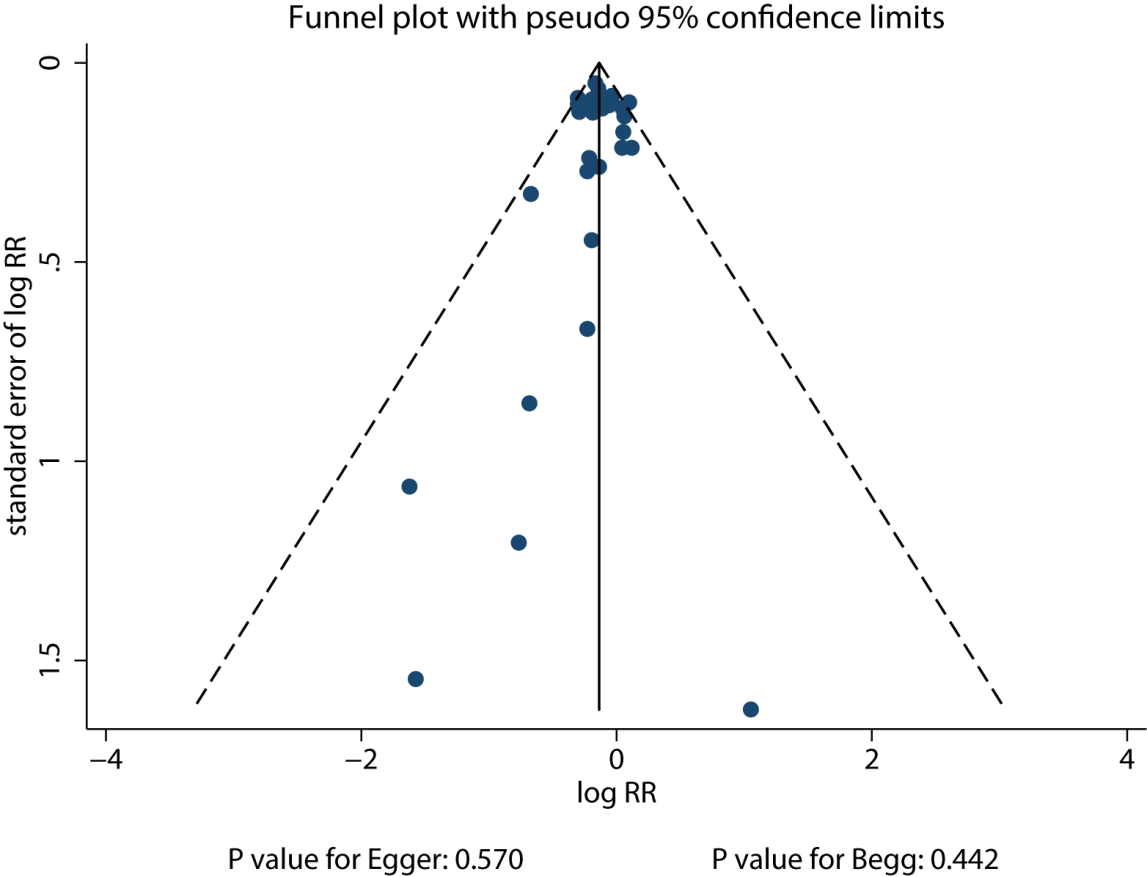


Figure S9. Funnel plot for all-cause mortality


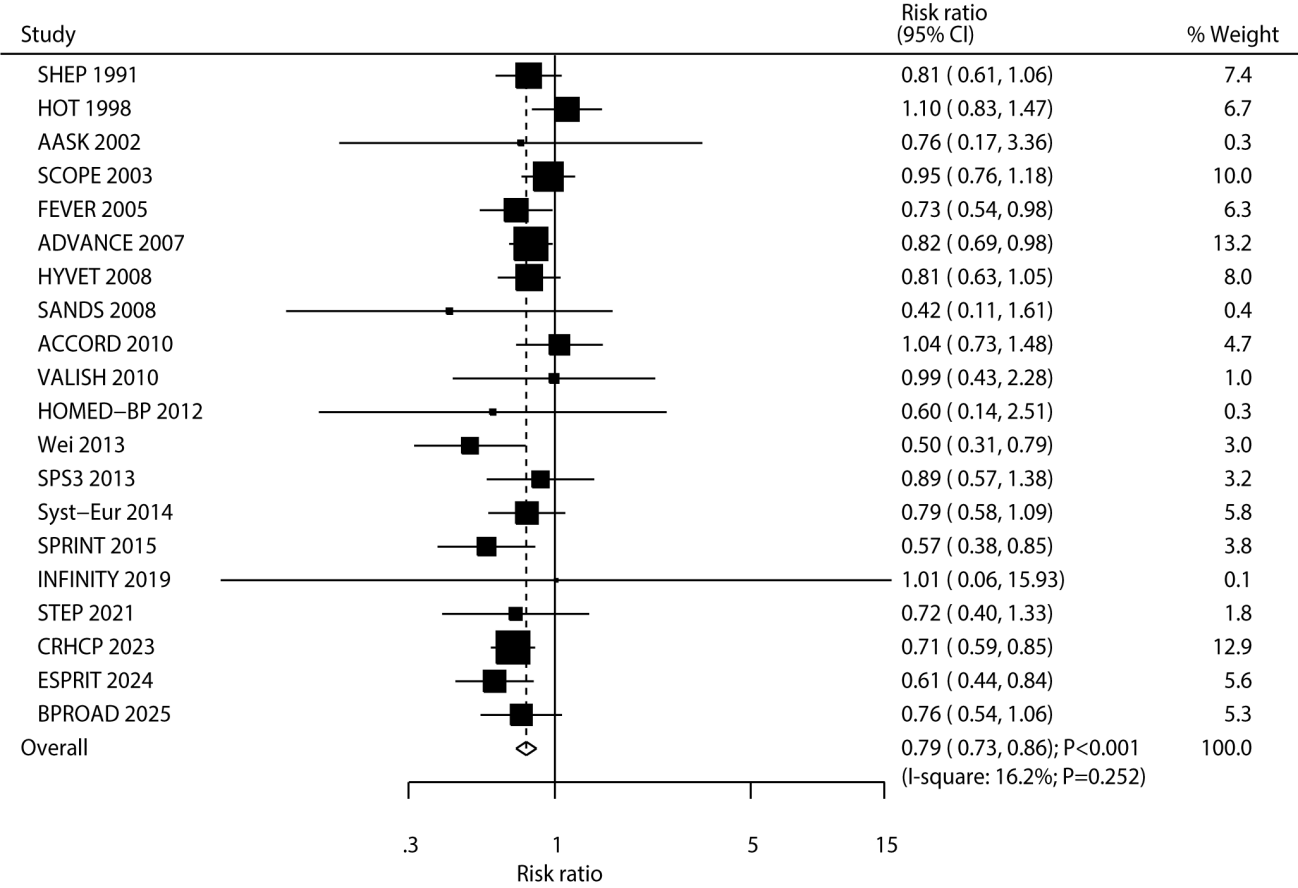


**Figure S10. Forest plot for the association between intensive versus standard blood pressure control and cardiac death.** This random-effects meta-analysis of randomized controlled trials is presented as a forest plot. Each horizontal line represents the 95% confidence interval (CI) for an individual study, and the square represents its point estimate (relative risk, RR). The area of each square is proportional to the study's weight in the meta-analysis. The dashed vertical line indicates the line of overall effect (pooled RR). The diamond at the bottom represents the overall pooled RR with its 95% CI. Heterogeneity among studies was quantified as I-square = 16.2%.


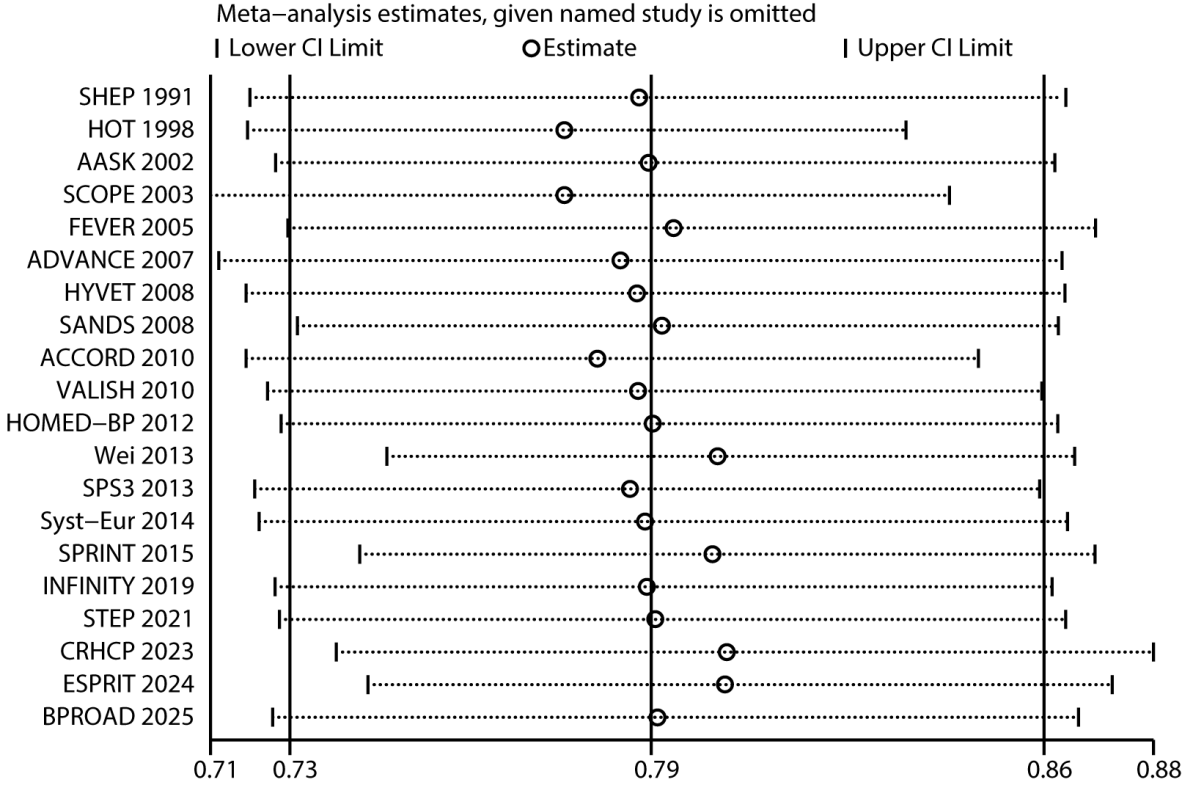


Figure S11. Intensive versus standard blood pressure control on the risk of cardiac death


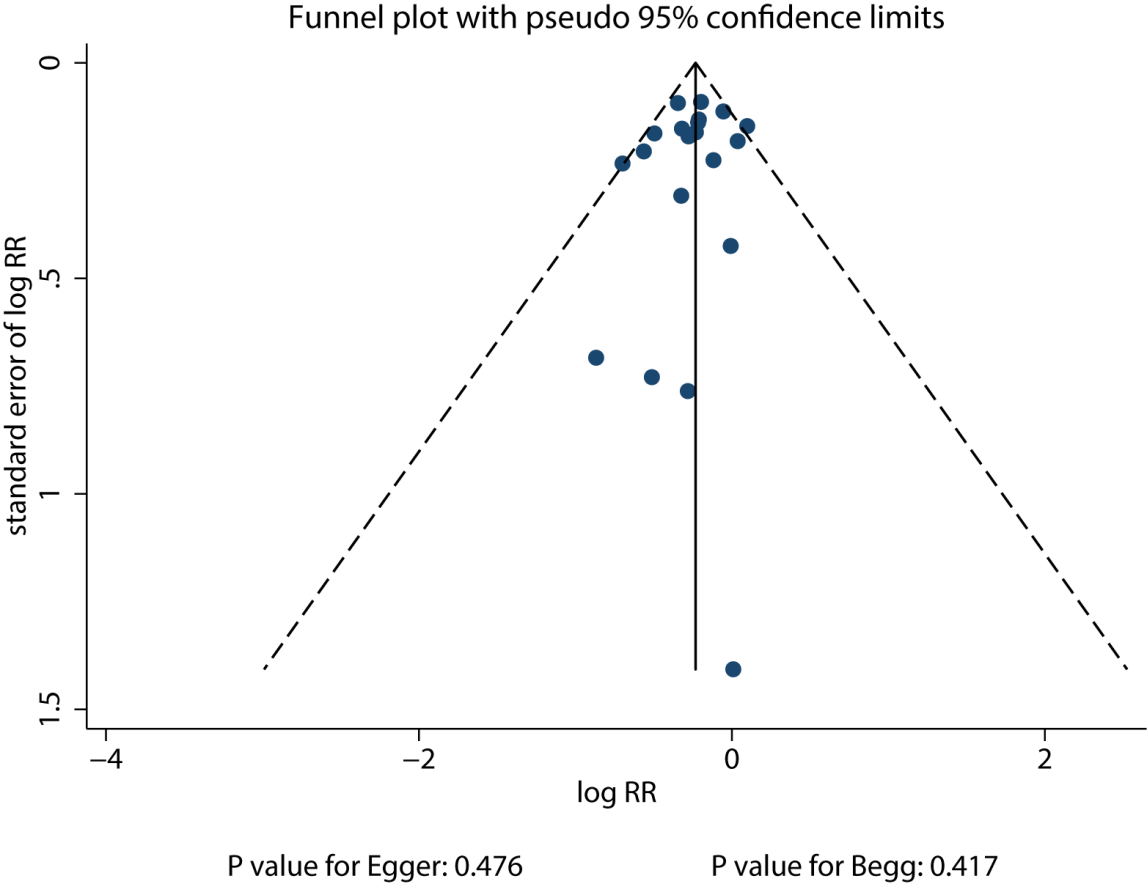


Figure S12. Funnel plot for cardiac death
